# *Salmonella enterica* Serotype Uganda Infection in New York City and Chicago^1^

**DOI:** 10.3201/eid1009.030713

**Published:** 2004-09

**Authors:** Roderick C. Jones, Vasudha Reddy, Laura Kornstein, Julio R. Fernandez, Faina Stavinsky, Alice Agasan, Susan I. Gerber

**Affiliations:** * Chicago Department of Public Health, Chicago, Illinois, USA;; † New York City Department of Health and Mental Hygiene, New York, New York, USA

**Keywords:** disease outbreaks, *Salmonella*, food handling, dispatch

## Abstract

Outbreaks associated with distinct strains of *Salmonella enterica* serotype Uganda, a rare serotype, occurred in New York City and Chicago during the summer of 2001. Both outbreaks were linked to eating ready-to-eat pork products. This serotype may emerge as a more frequent cause of human infections.

*Salmonella enterica* serotype Uganda (*S*. Uganda) is rarely isolated from humans in the United States. From 1993 to 2000, a median of 48 human isolates per year (*1*) and no foodborne disease outbreaks were reported (*2*). In August 2001, the New York City Department of Health and Mental Hygiene posted an alert on the Epidemic Information Exchange (Epi-X), a national, Web-based communications network for public health investigation and response (*3*), regarding its investigation of an outbreak of *S*. Uganda infections. Upon detection of a similar cluster in Chicago, the Chicago Department of Public Health notified New York City's health department, and the two agencies discussed methods and results.

The two cities' health departments interviewed case-patients with standardized questionnaires, conducted sanitary inspections of implicated food service establishments, interviewed and tested food workers, and analyzed food and environmental samples. Outbreak isolates were compared by molecular typing with pulsed-field gel electrophoresis (PFGE) with standardized PulseNet protocols (*4*).

## The Outbreaks

### New York City

**Figure 1:**
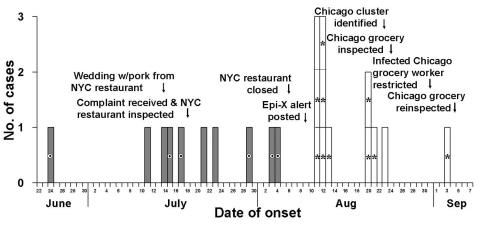
utbreak-associated *Salmonella enterica* serotype Uganda case-patients by date of illness onset, New York City (NYC) (shaded bars) and Chicago (clear bars), June–September, 2001. Onset date was not given for one NYC case-patient who attended an event at which pork from the implicated restaurant was served. Encircled bullet denotes those who recalled eating pork from the implicated NYC restaurant; asterisk denotes those who recalled eating carnitas from the implicated Chicago grocery.

On July 18, 2001, the New York City Department of Health and Mental Hygiene received a complaint of illness from a person who ate at a wedding celebration on July 14; *S*. Uganda was isolated from the stool of another wedding attendee. By early August, a distinct strain of *S*. Uganda had been isolated from 11 New York City residents with illness onsets occurring June 24–August 4 (Figure 1). All 11 case-patients were of Hispanic ethnicity, and 6 of 10 interviewed reported having eaten roast pork from a New York City restaurant in the 3 days before illness onset. Additionally, roast pork from that restaurant had been served at the wedding named in the initial consumer complaint. A sample of leftover roast pork from the wedding was positive for the same strain of *S*. Uganda as the one isolated from patients.

Preparation of the roast pork at the restaurant consisted of seasoning 30–40 lb of raw pork in buckets with salt, pepper, oregano, and garlic. The pork was subsequently cooked in an oven for 2 h at 500°F (260°C), then for 1 to 2 h more at 350°F (177°C). After being cooked, the pork was stored in a hot-holding unit at the front of the restaurant and cut into pieces upon request.

At the time of a sanitary inspection initiated by the consumer complaint, raw pork was held at inadequate temperatures at the restaurant, and thermometers were inadequately used during cooking and hot-holding. Potential sources of cross-contamination, surfaces and wiping cloths, were not properly sanitized. The same *S*. Uganda strain found in patients was isolated from a cooked pork sample collected from the restaurant on July 18. Raw pork sampled at that time was positive for *Salmonella*, but not *S*. Uganda. The restaurant was closed on August 6 and reopened on September 26, after food safety violations were corrected and pork samples were confirmed negative for *Salmonella*.

### Chicago

From August 22 to 23, 2001, the Chicago Department of Public Health received reports of four isolates of *S*. Uganda obtained from Chicago residents. By early September, 12 confirmed cases of *S*. Uganda infection had been identified in Chicago with stool collection dates from August 13 to September 6. All 12 patients were of Hispanic ethnicity, and each reported having eaten carnitas (fried pieces of pork) in the 3 days before illness onset. Carnitas were the only food item reported to have been eaten by all the patients. For 10 (83%) patients, the source of the carnitas was a Chicago grocery store. Of the two patients who did not identify that grocery, one had bought carnitas from a mobile lunch truck located outside of his workplace; the source of these carnitas could not be verified. The other patient was visiting from out of state and could not name the source of the carnitas.

Preparation of carnitas at the Chicago grocery consisted of cutting 90 lbs. of raw pork into 15 cm x 15 cm x 10 cm slabs, frying them in pork lard for 2.5 h, and adding salt and lemon juice 15 min before the end of frying. Cooked carnitas were then chopped, shredded into smaller pieces, and placed in a hot holding tray on top of the meat cooler. Using tongs, a customer would place carnitas into a Styrofoam container, then hand it to a butcher for weighing.

No *Salmonella* was isolated from samples of cooked carnitas, raw pork, the holding tray, or the serving tongs obtained from the Chicago grocery on August 24. *S*. Uganda was isolated from stool from 1 of 10 butchers. He reported having worked while having a mild gastrointestinal illness around August 15 and said he frequently ate carnitas from the Chicago grocery. The infected butcher was restricted from work until two consecutive stool specimens, collected 48 h apart, were negative for *Salmonella*. The workers and grocery manager were advised to avoid contact with food when sick.

The investigation of the Chicago grocery showed that processed meats were being stored at improper temperatures and that potential sources of cross-contamination were not controlled (i.e., cutting boards and utensils were inadequately sanitized, and the same scale was used for raw meats as well as cooked pork). Methods of preventing cross-contamination after cooking were stressed. Upon reinspection, the violations had been corrected.

**Figure 2:**
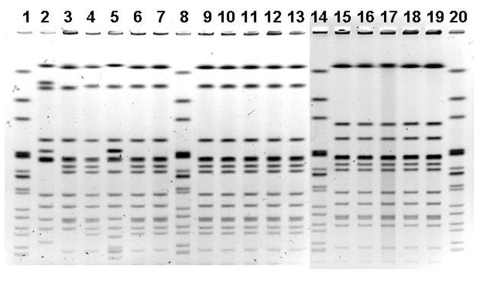
Pulsed-field gel electrophoresis profiles. Lanes 1, 8, 14, and 20, Centers for Disease Control and Prevention standard; lanes 2, 3, and 5, New York City nonoutbreak-associated clinical *Salmonella enterica* serotype Uganda (SU) case isolates; lanes 4, 6, and 9–13, New York City outbreak–associated clinical SU case isolates; lane 7, SU isolate obtained from leftover roast pork from wedding; lanes 15–19, Chicago outbreak-associated clinical SU case isolates.

Comparison of the outbreak isolates by PFGE showed a three-band difference (Figure 2). The sources of the pork implicated in the outbreaks were traced to two separate distributors; a more extensive traceback (i.e., to the packing-plant or farm source) was not conducted.

## Conclusions

We report temporally associated outbreaks of *S*. Uganda that likely resulted from eating contaminated ready-to-eat pork products. Including those obtained from outbreak patients in New York City and Chicago, 96 isolates of *S*. Uganda were obtained from human sources in the United States in 2001 (*1*). This number was the highest reported to the Centers for Disease Control and Prevention in the past decade. This finding is consistent with a previously reported trend toward increased incidence of this serotype; despite an overall downward trend in rates of *Salmonella* infections in the United States, from 1987 to 1997, *S*. Uganda had the eighth highest annual percentage increase in human isolates during this period (*5*). The outbreaks and these data may signal the emergence of *S*. Uganda as a serotype more frequently associated with human infections in the United States.

Despite the rare occurrence of *S*. Uganda and the temporal association and epidemiologic similarity of the outbreaks, molecular analysis of the outbreak isolates suggested that the New York City and Chicago illnesses were not linked to a common source. The outbreak isolates were also distinguishable from *S*. Uganda clinical isolates posted on the PulseNet Web board in 2001, which indicates a low likelihood that a multijurisdictional common-source *S*. Uganda outbreak had occurred. While this serotype is not among the 10 most common *Salmonella* serotypes found in swine carcasses or raw ground pork (*6*), it is routinely isolated from swine sources (*1*). Random sample of *S*. Uganda isolates obtained from swine in 2001 from the National Veterinary Services Laboratory were distinguishable from the outbreak isolates by PFGE. These findings imply heterogeneity in the PFGE patterns of *S*. Uganda isolates and suggest that molecular analysis is a useful tool to assess the relatedness of different strains for outbreak investigations.

Of 357 *Salmonella* foodborne disease outbreaks reported from 1993 to 1997, the vehicle of transmission was classified as pork in 4 (1.1%) outbreaks, a proportion lower than that of beef (3.9%), chicken (1.6%), and turkey (1.6%) (*7*). Nonetheless, *Salmonella* organisms may be present on an estimated 5%–10% of market hog carcasses after slaughter (*8*), and the outbreaks described here highlight the potential for *Salmonella* transmission to humans through cooked pork. First, the implicated establishments in New York City and Chicago both cooked large quantities of pork at one time and held the ready-to-eat product in a hot-holding unit; improper holding temperatures could allow growth of any *Salmonella* organisms that survived cooking. Second, food workers cut or weighed the ready-to-eat product before sale at both establishments, and such contact introduces another potential source of contamination. Third, both investigations cited sources of potential cross-contamination of cooked product by raw meat. In Chicago, butchers who handled raw meat also served customers, and scales were used to weigh raw meat as well as cooked carnitas. Use of the same knives, cutting boards, or other utensils could have contributed to contamination of the ready-to-eat product in either outbreak. *S*. Uganda was isolated from the stool of one of the Chicago butchers; whether he contributed to disease transmission is unclear. However, infected food workers who do not practice proper hygiene could have played a part in these outbreaks. Previous reports of pork-associated salmonellosis outbreaks have noted the importance of adequately reheating cooked pork if time lapses between preparation and consumption (*9**,**10*). Had the pork implicated in the outbreaks been reheated to an internal temperature of 165°F (74°C), the *S*. Uganda infections likely could have been prevented.

A common source was not implicated in the New York City and Chicago outbreaks, but the experience underscores the potential for *Salmonella* transmission through contaminated ready-to-eat pork products, the importance of PFGE in *Salmonella* outbreak investigations even when the serotypes involved are rare, and the utility of the Epi-X system to alert local health departments and facilitate data-sharing.
